# Histotripsy in tumor therapy: advances in non-thermal mechanical ablation, immune modulation, and clinical translation

**DOI:** 10.3389/fimmu.2026.1858144

**Published:** 2026-10-12

**Authors:** Yangyang Ma, Xinan Long, Jiangpeng Cheng, Lizhi Niu

**Affiliations:** 1 Central Laboratory, Guangzhou Fuda Cancer Hospital, Guangzhou, China; 2 Department of Oncology, Guangzhou Fuda Cancer Hospital, Guangzhou, China

**Keywords:** boiling histotripsy, cavitation, clinical translation, focused ultrasound, histotripsy, immunogenic cell death, microtripsy, tumor ablation

## Abstract

Histotripsy is an emerging non-invasive focused-ultrasound technology that mechanically disrupts tissue through controlled bubble activity rather than sustained thermal coagulation. This review distinguishes shock-scattering cavitational histotripsy, intrinsic threshold histotripsy, and boiling histotripsy and relates their pulse regimes, bubble dynamics, tissue effects, monitoring strategies, and evidence maturity. Mechanical fractionation can expose tumor antigens and damage-associated molecular patterns, but immune outcomes vary by submodality, dose, tumor model, treated fraction, and sampling time; no standardized head-to-head evidence establishes a superior immunological approach. Early prospective liver studies demonstrate high technical feasibility and encouraging local control, whereas post-market cohorts also reveal targeting limitations and uncommon but clinically important vascular, thrombotic, and renal complications. Pancreatic investigation remains ongoing, while the renal CAIN study is completed with registry results and a peer-reviewed first-patient report. A clinically useful dosimetry framework should connect delivered acoustic parameters with *in situ* cavitation, tissue disruption, imaging or acoustic surrogates, and delayed oncologic and immune endpoints. Histotripsy should currently be viewed as a complementary local therapy with a distinctive mechanical mechanism rather than as a proven replacement for established ablation. Standardized reporting, independently adjudicated safety data, and prospective comparative trials are needed to define its durable oncologic and immunological value.

## 1 Introduction and developmental context

Histotripsy is a focused-ultrasound ablation strategy designed to destroy tissue by controlled mechanical effects rather than sustained thermal coagulation. The first renal-tissue report using the term histotripsy appeared in 2006, followed by mechanistic work defining cavitation-cloud formation and later by distinct implementations based on shock-scattering histotripsy, intrinsic threshold histotripsy, and boiling histotripsy (1–4). These approaches share low-duty-cycle focused-ultrasound delivery and mechanically disruptive bubble activity, but they should not be treated as technically or biologically interchangeable.

Interest in oncology has expanded because mechanical tissue fractionation can be delivered without a skin puncture, can be visualized during treatment, and may release tumor antigens and damage-associated molecular patterns (DAMPs). Preclinical studies have described local immune remodeling and, in selected models, inhibition of untreated tumors (5, 6). These findings remain model- and protocol-dependent; they do not yet establish a reproducible systemic immune benefit in patients.

Translation progressed from a first human study in benign prostatic enlargement in 2018 to the THERESA liver-tumor study, the 2023 U.S. Food and Drug Administration *De Novo* classification of the Edison System, and subsequent prospective and real-world liver cohorts (7–10). Current oncologic evidence is summarized in **Table 1**. This review therefore evaluates histotripsy by explicitly linking subtype, delivered dose, monitoring method, tissue response, immune observations, and evidence maturity.

**Table 1:** Current oncologic evidence for histotripsy.

Study	Tumor/model	Evidence	Sample	Subtype	Treatment	Main finding	Limitation/status
Qu et al., 2020 (6)	Melanoma; mouse	Preclinical	NR	Cavitational histotripsy	Histotripsy ± anti-PD-1	Local and abscopal immune responses; enhanced checkpoint response	Model-specific; no clinical validation
Pahk et al., 2019 (29)	Solid tumor models	Preclinical	NR	Boiling histotripsy	Partial mechanical ablation	ICD-associated signals and TME modulation	Protocol and model dependent
Thim et al., 2024 (30)	Melanoma; mouse	Preclinical	NR	Boiling histotripsy	Sparse-scan ablation	Antigen-dependent DC activation and distant control	Transient antigen signal; animal model
Stepanechko et al., 2025 (31)	Pancreatic cancer; mouse	Preclinical	NR	Boiling histotripsy	BH ± anti-CD40	Neither enhanced nor negated anti-CD40 activity under the tested protocol	Demonstrates context dependence
McGinnis et al., 2025 (54)	Melanoma; mouse	Dose-response	12-13/group	Intrinsic threshold histotripsy	8, 20, 40, 100 ppl	40 ppl: best tumor control and greatest CD45+/CD8+ infiltration	Preclinical; no clinical immune dose
Worlikar et al., 2026 (55)	Orthotopic HCC; rat	Dose-response	NR	Intrinsic threshold histotripsy	20, 50, 100, 200 ppl	Intermediate dose optimized stage-specific response; 200 ppl not best	Preclinical; PRF covaried with ppl
THERESA, 2022 (8)	Liver tumors	First-in-human	8 patients	Cavitation histotripsy; subtype not reported	Image-guided partial/complete treatment	100% technical endpoint; no device-related SAE	Small, single-arm feasibility
#HOPE4LIVER, 2024 (10)	Liver tumors	Prospective multicenter	44 patients; 49 tumors	Cavitation histotripsy; subtype not reported	Robotic US-guided treatment	95% technical success; 7% major complications	Single arm; small tumors; 30-day endpoint
#HOPE4LIVER, 2025 (60)	Liver tumors	1-year follow-up	47 patients; 52 tumors	Cavitation histotripsy; subtype not reported	Robotic US-guided treatment	1-year local control 63.4% primary/90% *post hoc*	Imaging learning curve; six serious ADEs <=30 d
Wehrle et al., 2025 (61)	Liver tumors	Real-world safety	230 with safety data	Cavitation histotripsy; subtype not reported	Post-clearance practice	5.2% any complication; 1.3% major, recorded deaths from progression	Retrospective; attribution limitations
Wehrle et al., 2025 (62)	Liver tumors	Multicenter LTC	47 patients; 91 tumors	Cavitation histotripsy; subtype not reported	Complete-treatment intent	95% nonviable at 30 d; 94% primary efficacy at 90 d	Short follow-up; systemic therapy confounding
Breuer et al., 2026 (16)	Liver tumors	Safety case series	139 patients	Cavitation histotripsy; subtype not reported	Routine practice	4 serious vascular injuries (2.9%); 3 embolizations; 1 death	Preliminary, multicenter case series
Poe et al., 2026 (64)	Liver tumors	Safety case series	146 procedures	Cavitation histotripsy; subtype not reported	Routine practice	9 HAKI cases (6.2%); larger overlapping actual volumes	Preliminary; risk thresholds undefined
Worlikar et al., 2026 (17)	Perivascular liver tumors	High-risk subset	21 patients	Cavitation histotripsy; subtype not reported	Zone abutting/encasing large vein	8 bland PVT cases (38%); improved/resolved with anticoagulation	Enriched subset; not general incidence
Wah et al., 2025 (51)	Renal cell carcinoma	First-patient report	1 patient	Cavitation histotripsy; subtype not reported	CAIN protocol	Successful treatment with 12-month follow-up	Case report only
CAIN, NCT05432232 (49)	Primary solid renal tumors	Completed registry	20 enrolled	Cavitation histotripsy; subtype not reported	Prospective single-arm pilot	Results posted Dec 2, 2025	Full peer-reviewed cohort unavailable at cutoff
GANNON, NCT06282809 (46)	Pancreatic adenocarcinoma	Ongoing registry	Target 50	Cavitation histotripsy; subtype not reported	Prospective safety/feasibility	Recruiting; no results posted	Investigational pancreatic use

ADE, adverse device-related effect; BH, boiling histotripsy; CAIN, Cavitation of Kidney Tumors study; DC, dendritic cell; HAKI, histotripsy-associated acute kidney injury; HCC, hepatocellular carcinoma; ICD, immunogenic cell death; LTC, local tumor control; NR, not reported; PD-1, programmed cell death protein 1; ppl, pulses per location; PRF, pulse-repetition frequency; PVT, portal vein thrombosis; SAE, serious adverse event; TME, tumor microenvironment; US, ultrasound.

## 2 Physical mechanisms, tissue effects, and monitoring

### 2.1 Histotripsy submodalities

Shock-scattering cavitational histotripsy commonly uses several-cycle, high-amplitude pulses. A bubble initiated during the early cycles backscatters an incident positive shock; phase inversion can reinforce the rarefactional pressure and extend the cloud toward the transducer. The often-cited 5-20-cycle range at 1 MHz is representative of the foundational experimental system, not a universal prescription (2).

Intrinsic threshold histotripsy uses approximately 1-2-cycle pulses with peak-negative pressure above the medium’s intrinsic cavitation threshold. Across water-rich tissues and frequencies from 345 kHz to 3 MHz, the 50% cavitation-probability threshold was approximately 24.7-30.6 MPa in one experimental series (3). This threshold describes bubble-cloud initiation. It should not be conflated with the dose required for complete tissue fractionation, which depends on repeated exposures, focal spacing, and tissue composition.

Boiling histotripsy uses millisecond-scale pulses with nonlinear shocks. Localized shock heating creates a vapor cavity, after which vapor-bubble motion, shock scattering, cavitation, and acoustic streaming mechanically fractionate tissue. Although heat initiates the vapor cavity, the intended bulk lesion is mechanical rather than conventional thermal coagulation (11, 12). Representative parameters and evidence maturity are compared in **Table 2** and **Figure 1**.

**Table 2:** Technical distinctions among major histotripsy submodalities.

Subtype	Pulse regime	Cloud initiation	Representative parameters	Bubble/tissue pattern	Monitoring	Evidence maturity
Shock-scattering cavitational histotripsy (2)	Several-cycle, nonlinear pulse	Sparse initiating bubble backscatters positive shock; inverted wave reinforces rarefaction	5–20 cycles at 1 MHz in foundational experiments	Cloud elongates toward transducer	B-mode, backscatter, Doppler, emissions	Extensive preclinical; mechanism established
Intrinsic threshold histotripsy (3)	Very short pulse, usually 1–2 cycles	Peak-negative pressure directly exceeds intrinsic threshold	Approx. 24.7-30.6 MPa across tested water-rich tissues/frequencies	Compact cloud in threshold-crossing focal volume	B-mode, passive cavitation detection, emissions	Extensive preclinical evidence; relevance to current cavitational systems, although device-specific subtype is often not reported
Boiling histotripsy (11)	Millisecond nonlinear-shock pulse; low duty cycle	Shock heating initiates vapor cavity; bubble motion, cavitation, and streaming fractionate tissue	Often 1–10 ms; system- and tissue-dependent	Vapor cavity with adjacent mechanical fragmentation	B-mode boiling/cavitation signatures; post-treatment imaging	Preclinical oncology and non-oncology; limited clinical translation

Parameter ranges are representative of cited experimental systems and are not universal treatment prescriptions. B-mode, brightness mode; MPa, megapascal; US, ultrasound.

**Figure 1:**
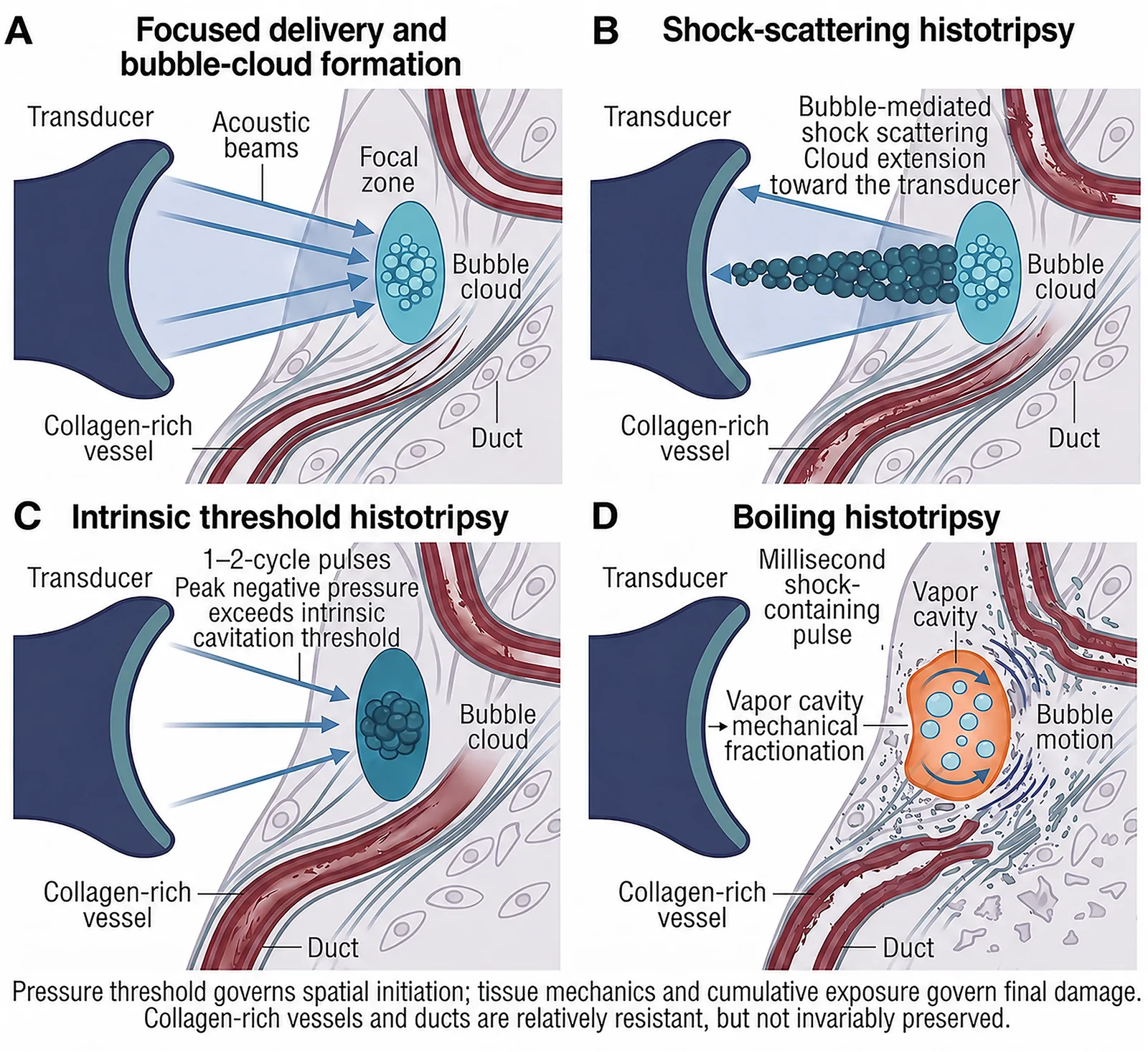
Mechanistic spectrum and tissue selectivity of histotripsy. **(A)** A concave focusing transducer concentrates acoustic energy within a defined focal zone, where controlled bubble activity mechanically fractionates tissue. **(B)** Shock-scattering cavitational histotripsy uses several-cycle nonlinear pulses; an initiating bubble scatters an incident positive shock, and the resulting inverted rarefaction promotes bubble-cloud extension toward the transducer. **(C)** Intrinsic threshold histotripsy uses approximately 1–2-cycle pulses whose peak-negative pressure directly exceeds the intrinsic cavitation threshold within the focal region. **(D)** Boiling histotripsy uses millisecond shock-containing pulses to initiate a vapor cavity, followed by bubble motion and mechanical fractionation. Pressure-threshold gating governs spatial initiation, whereas tissue mechanics and cumulative exposure govern final damage. Collagen-rich vessels and ducts are relatively resistant, but not invariably preserved. The drawings are mechanistic schematics based on foundational studies (2, 3, 11, 12) and should not be interpreted as head-to-head evidence of subtype-specific structural preservation.

### 2.2 Boundary formation and tissue susceptibility

Relatively sharp lesion margins arise primarily because cavitation is threshold-gated: bubble activity is concentrated where the local waveform exceeds the relevant cloud-initiation condition. The pressure field can therefore confine treatment more tightly than a simple statement about tissue stiffness would imply. After initiation, however, bubble expansion, collapse, and accumulated structural damage depend on tissue mechanics, collagen content, interfaces, perfusion, and the number and spatial overlap of pulses (13–15).

Parenchymal tissue is generally more susceptible to homogenization than collagen-rich vessels and ducts, but this is relative resistance rather than guaranteed structure preservation. Recent clinical reports of hemorrhage, pseudoaneurysm, portal-vein thrombosis, and other off-target effects demonstrate that an apparently sharp parenchymal boundary does not eliminate anatomy-specific risk (16, 17). Histotripsy should therefore be described as spatially confined but not universally structure-sparing.

### 2.3 Imaging guidance and treatment monitoring

B-mode ultrasound depicts the bubble cloud as a dynamic hyperechoic region and remains the principal real-time targeting and feedback method. Quantitative backscatter can decrease as cellular structures are fractionated; bubble-induced color Doppler tracks residual-nuclei motion after cloud collapse; and cavitation-emission mapping can localize activity or estimate near-complete treatment. Each surrogate is sensitive to tissue type, insonation geometry, and acquisition settings and therefore requires calibration against histology or validated post-treatment imaging (18–21).

MRI monitoring is experimental rather than routine for current hepatic systems. Gradient timing can make gradient-echo signal intensity sensitive or insensitive to transient cavitation, while post-treatment T2-weighted and contrast-enhanced imaging can characterize the evolving treatment zone (22). Transcranial MR-guided histotripsy has been demonstrated preclinically (23).

CT-based navigation may supplement ultrasound when targets are poorly visualized. In seven porcine liver treatments, cone-beam CT-guided robotic registration achieved complete pseudotumor coverage and a center-to-center targeting error of 3.8 ± 1.3 mm (24, 25). These are preclinical precision data and should not be generalized to routine human performance.

### 2.4 Non-oncologic technical background

Non-oncologic studies illustrate how mechanism and dose can be matched to different tissue targets. Intrinsic threshold histotripsy restored or substantially increased venous flow in 13 of 14 pigs with acute deep-vein thrombosis, with an average treatment time of 16 minutes per centimeter of thrombus and no observed vessel-wall injury or pulmonary embolism at two weeks (26). *In vitro*, a combined boiling- and cavitational-histotripsy strategy produced 20 mL of aspiratable hematoma lysate in 17.5 minutes, with different lesion regularity and efficiency for the two submodalities (27). Three-dimensional echocardiography-guided cavitational histotripsy also cut mitral-valve chordae in beating sheep hearts in 20 ± 9 minutes without significant mitral regurgitation in that experiment (28). These results provide technical lessons, not evidence of established non-oncologic indications.

## 3 Histotripsy as a potential immunomodulatory platform

### 3.1 DAMP release, antigen exposure, and modality dependence

Mechanical lysis can expose tumor-derived antigens and release DAMPs, including HMGB1, ATP, and calreticulin-associated signals. Cavitational histotripsy studies in melanoma and other models have reported spatially and temporally heterogeneous cell death, immune infiltration, and abscopal-like responses (5, 6). Boiling histotripsy studies have separately reported immunogenic-cell-death signatures, antigen acquisition by dendritic cells, and distant tumor control under selected sparse-scan protocols (29, 30).

These findings should not be pooled as a single modality-independent immune phenotype. Shock-scattering histotripsy, intrinsic threshold histotripsy, and boiling histotripsy differ in waveform, bubble dynamics, residual tissue structure, treated fraction, and dose. A pancreatic model in which a single boiling-histotripsy exposure neither enhanced nor negated anti-CD40 activity further illustrates context dependence (31). No standardized head-to-head study has established that one histotripsy subtype produces superior DAMP release, immunogenic cell death, or systemic immunity.

### 3.2 Antigen presentation and tumor-microenvironment remodeling

A plausible sequence is uptake of mechanically released material by antigen-presenting cells, migration to draining lymph nodes, and priming of tumor-reactive T cells. Direct demonstration of every step remains limited, although boiling-histotripsy melanoma experiments showed transient antigen acquisition and antigen-dependent dendritic-cell activation, and cavitational studies reported increased CD8-positive T-cell infiltration (30, 32, 33). Complementing these cellular studies, Tang et al. demonstrated histotripsy-triggered release of the tumor-associated antigen HER2 into the extracellular compartment in breast-cancer cell and tumor models, with greater free HER2 release at higher tested doses (34). **Figure 2** therefore labels the local cascade as preclinically supported while depicting systemic or abscopal effects as uncertain.

**Figure 2:**
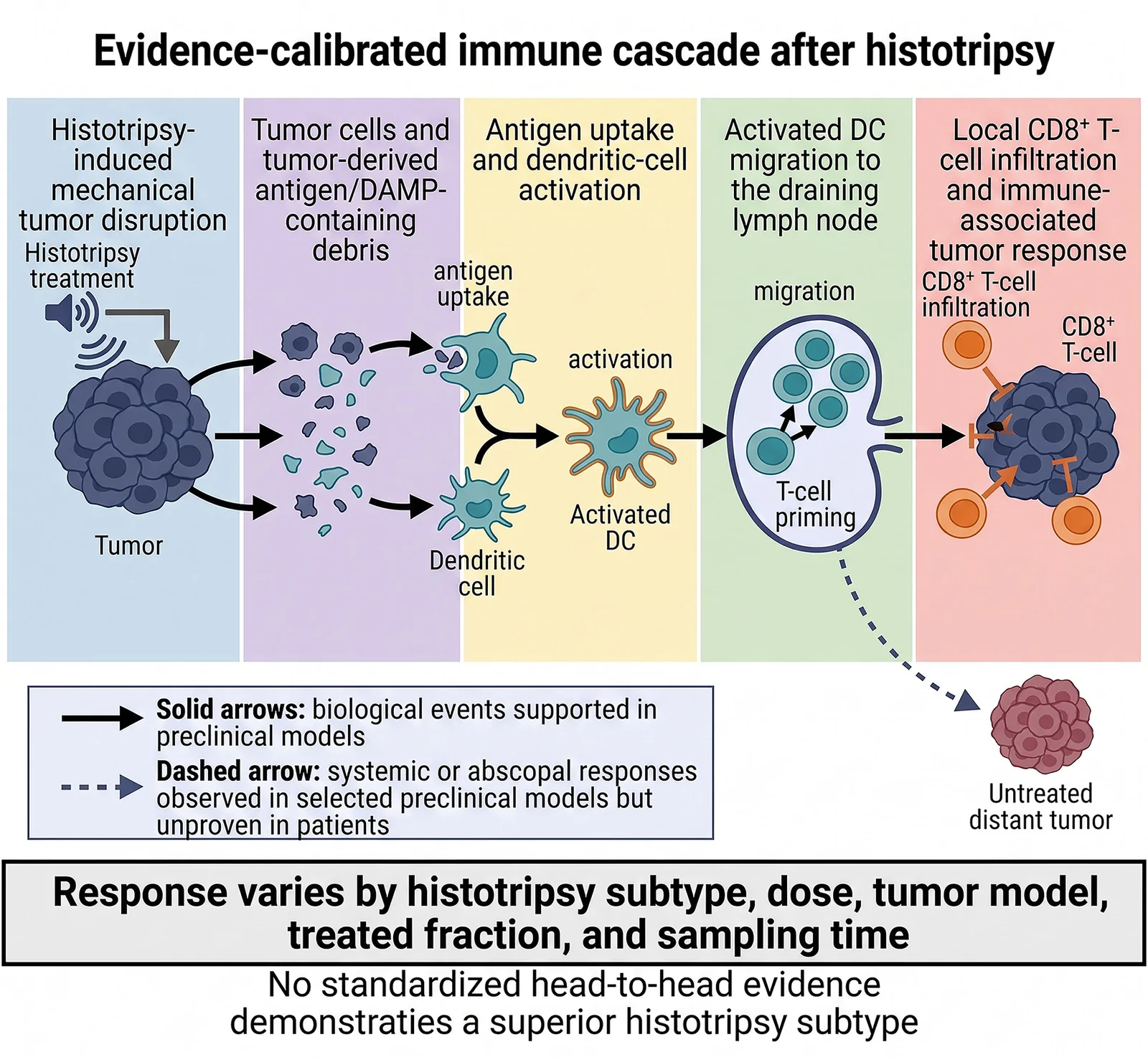
Evidence-calibrated immune cascade after histotripsy. Mechanical tumor disruption can generate tumor-cell and tumor-derived antigen/DAMP-containing debris, followed by antigen uptake, dendritic-cell activation, migration to the draining lymph node, T-cell priming, and local CD8+ T-cell infiltration in preclinical models (5, 6, 29, 30, 32–34). Solid arrows denote biological events supported in preclinical studies. The dashed arrow denotes systemic or abscopal responses observed in selected preclinical models but not yet established in patients. Responses vary with histotripsy subtype, acoustic dose, tumor model, treated fraction, and sampling time (31, 35, 36); no standardized head-to-head evidence demonstrates that one subtype produces a superior immune response. DAMP, damage-associated molecular pattern; DC, dendritic cell.

Local remodeling is similarly heterogeneous. Changes in hypoxia signaling, cytokines, macrophages, and cytotoxic lymphocytes vary with tumor model, ablation fraction, dose, and sampling time (33–36). Consequently, immune biomarkers should be interpreted with the delivered acoustic protocol and tissue-damage phenotype rather than with the label ‘histotripsy’ alone.

### 3.3 Systemic responses and combination therapy

Untreated-tumor regression has been observed in selected animal models and isolated clinical observations, but frequency and clinical relevance are unknown (6, 37). The stronger translational hypothesis is that histotripsy may function as a local priming or sensitizing intervention when paired with systemic treatment, not that local treatment reliably produces stand-alone systemic control.

Original preclinical studies support combinations with immune-checkpoint blockade or CD40 stimulation in selected settings (6, 38). A peer-reviewed 2026 neuroblastoma study reported that histotripsy-initiated immune responses synergized with chemotherapy in a murine model (39). Optimal sequencing, treated fraction, and partner therapy remain unresolved.

## 4 Preclinical evidence across tumor models

### 4.1 Why liver tumors became the leading indication

The liver became the principal translational target because it is a large parenchymal organ with an extensive preclinical safety literature, can often be visualized with ultrasound, contains tumors for which heat-sink effects and percutaneous access may be clinically relevant, and can be treated with robotic respiratory-motion compensation. Early porcine studies demonstrated image-guided cavitational lesion creation and chronic healing, while murine studies enabled simultaneous assessment of local destruction and immunity (40–42). These advantages, together with commercial system development and the unmet need in unresectable disease, supported the first oncologic trials and regulatory pathway.

The liver is not an acoustically simple target. Ribs, lung, bowel gas, subcutaneous depth, respiratory motion, lesion size, and proximity to portal or hepatic veins can restrict access or increase risk. Fibrotic tumors such as cholangiocarcinoma can require higher treatment exposure than softer tissues (15). The liver evidence base should therefore not be generalized automatically to pancreas, kidney, or other organs.

### 4.2 Pancreatic, renal, and other solid tumors

Pancreatic studies include cavitational histotripsy in orthotopic porcine and subcutaneous tumor models and boiling histotripsy in murine immunology experiments (31, 43–45). Bowel gas, limited acoustic windows, dense stroma, and adjacent bowel or vascular structures remain major constraints. The prospective GANNON feasibility study (NCT06282809) is recruiting, with no results posted as of August 27, 2026 (46).

Renal histotripsy has progressed from rabbit and porcine studies to early human evaluation (12, 47, 48). The CAIN study (NCT05432232) is completed and registry results have been posted; the peer-reviewed literature currently includes the protocol and a first-patient report with 12-month follow-up rather than a full peer-reviewed cohort publication (49–51). This distinction is important when reporting technical success and adverse events. Other tumor applications, including neuroblastoma, osteosarcoma, and spontaneous canine soft-tissue sarcoma, remain preclinical or veterinary-translational (32, 52, 53).

### 4.3 Dose-response and combination design

Histotripsy dose cannot be represented by a single universal quantity. Minimum reporting should include submodality, center frequency, pulse length and waveform, estimated *in situ* peak pressures, pulse-repetition frequency, pulses per focal location, focal-point spacing, scan trajectory, treated fraction, overlapping treatment volumes, and whether aberration or motion correction was used. Biological evaluation should distinguish immediate cellular disruption from residual architecture, delayed local control, and immune endpoints.

Two recent cavitational-histotripsy studies demonstrate that greater exposure does not necessarily maximize immune or tumor outcomes. In B16F10 melanoma, 40 pulses per location produced the greatest tumor control and the largest day-7 CD45-positive and CD8-positive infiltrates among 8, 20, 40, and 100 pulses per location (54). In orthotopic rat hepatocellular carcinoma, 100 pulses per location produced the strongest late-stage immune infiltration and regression among 20, 50, 100, and 200 pulses per location, whereas 200 pulses represented overtreatment and did not produce the best outcome (55). These preclinical results support non-monotonic dose optimization but do not define a clinical immune dose.

## 5 Comparison with established local therapies and clinical positioning

Histotripsy differs from radiofrequency ablation and microwave ablation by using extracorporeal, non-thermal mechanical fractionation rather than probe-based heating; from thermal high-intensity focused ultrasound by its intended mechanical rather than coagulative endpoint; and from irreversible electroporation by avoiding percutaneous electrode placement and producing tissue homogenization rather than primarily membrane electroporation (56–59). Potential advantages include non-invasive delivery, reduced dependence on thermal conduction, and real-time visualization of bubble activity. Limitations include the need for an acoustic window, motion and aberration management, treatment-volume constraints, incomplete clinical dose standardization, and newly recognized vascular and renal safety signals.

No randomized or adequately controlled head-to-head study currently establishes superiority, equivalence, or lower morbidity relative to established ablation. Clinical selection should therefore be scenario-based: mature thermal modalities remain standards where percutaneous access and heat delivery are acceptable; irreversible electroporation may be considered where its established indications and structural effects are desired; and histotripsy may be considered within its cleared liver indication or a clinical trial when non-invasive delivery and a suitable acoustic window are present. **Figure 3** provides a descriptive scenario matrix rather than a ranking.

**Figure 3:**
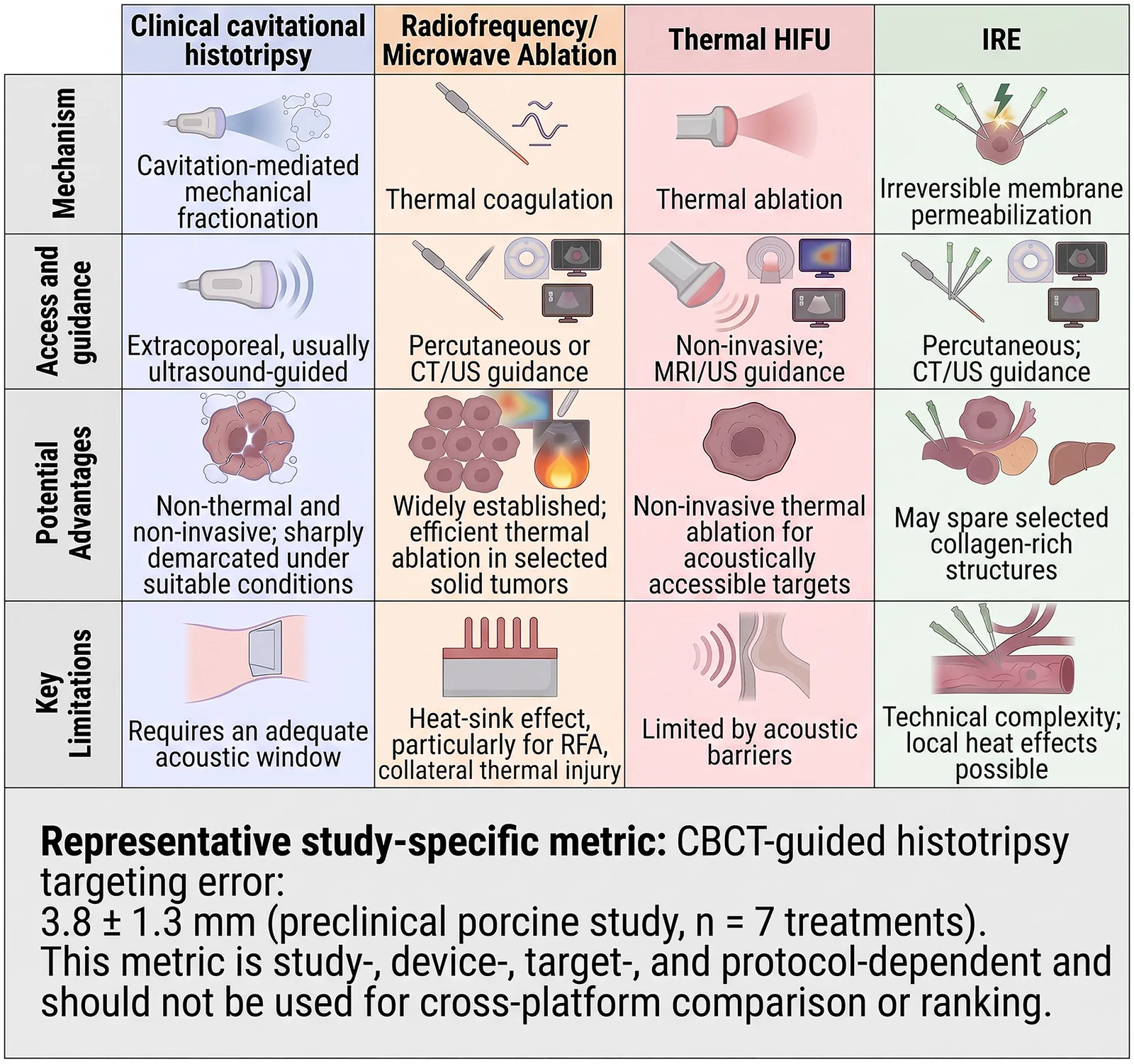
Scenario-based comparison of clinical cavitational histotripsy and established local tumor therapies. The matrix compares mechanism, access and image guidance, potential advantages, and key limitations without implying therapeutic superiority (56–59). The histotripsy column refers primarily to currently deployed clinical cavitational systems and does not represent boiling histotripsy. The representative CBCT-guided targeting error of 3.8 ± 1.3 mm was measured in seven treatments in a preclinical porcine model (24). This value is study-, device-, target-, and protocol-dependent and must not be used for cross-platform comparison or ranking. Radiofrequency and microwave ablation are distinct thermal techniques but are grouped here for a compact scenario-based comparison. No randomized head-to-head oncologic trial currently establishes superiority of histotripsy over established local therapies (66). CBCT, cone-beam computed tomography; CT, computed tomography; HIFU, high-intensity focused ultrasound; IRE, irreversible electroporation; MRI, magnetic resonance imaging; US, ultrasound.

## 6 Clinical translation and early clinical evidence

### 6.1 THERESA and #HOPE4LIVER

The THERESA feasibility study treated eight patients with primary or metastatic liver tumors and achieved its technical endpoint in all treated patients, without device-related serious adverse events in that small cohort (8). The prospective #HOPE4LIVER pivotal study subsequently reported 95% technical success (42/44 evaluable lesions) and procedure-related major complications in 7% (3/44 participants) at 30 days (10). These single-arm studies established feasibility but were not designed to compare oncologic efficacy with other local therapies.

At one year, 47 patients with 52 tumors had local control of 63.4% under the prespecified sequential imaging assessment and 90% under a *post hoc* experienced-reader assessment (60). Six serious adverse device-related effects occurred within 30 days; one nonserious biloma was reported after 30 days. One-year overall survival was 73.3% for hepatocellular carcinoma and 48.6% for metastatic disease (60). The discrepancy between primary and *post hoc* imaging assessments underscores a learning curve and the need for standardized response criteria.

### 6.2 Post-trial clinical outcomes and complications

Post-clearance cohorts provide broader but still non-randomized evidence. In a 230-patient safety analysis, 12 patients (5.2%) experienced complications of any grade and 3 (1.3%) had major complications recorded as deaths from disease progression in patients treated with palliative intent; these should not be described as proven device-caused deaths (61). A four-center cohort of 47 patients and 91 tumors intended for complete treatment reported 95% nonviability at 30 days and 94% primary efficacy at 90 days, with three complications (62). A single-center first-year cohort showed that when lesions were nonviable on postoperative day 1, all evaluable lesions remained nonviable at day 30, but only 74% of lesions intended for complete treatment were nonviable on day 1, emphasizing targeting and learning-curve limitations (63).

Recent reports also define safety boundaries. Serious vascular injury occurred in 4 of 139 patients (2.9%) across four centers, with three embolizations and one death (16). Histotripsy-associated acute kidney injury was reported after 9 of 146 procedures (6.2%) and was associated with larger cumulative or overlapping actual treatment volumes (64). In an enriched high-risk subset whose expected treatment zones abutted or encased a large portal or hepatic vein branch, bland portal-vein thrombosis occurred in 8 of 21 patients (38%) and improved or resolved with anticoagulation (17). The latter percentage must not be presented as a general-population incidence. **Figure 4** summarizes the developmental timeline and presents these clinical estimates with their original denominators.

**Figure 4:**
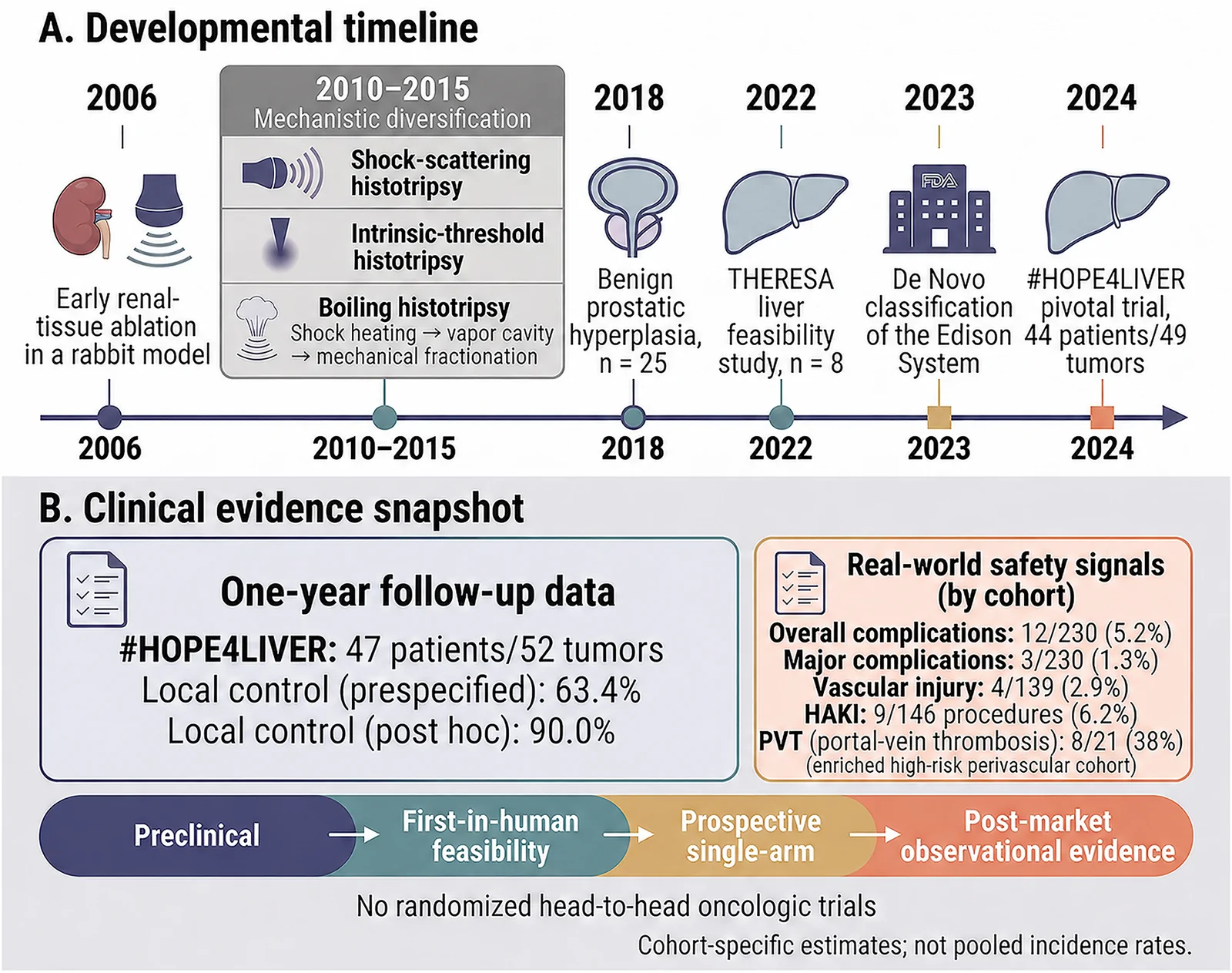
Developmental timeline and clinical evidence snapshot. **(A)** Key milestones extend from early rabbit renal-tissue ablation in 2006 (1) and mechanistic diversification into shock-scattering cavitational histotripsy, intrinsic threshold histotripsy, and boiling histotripsy (2–4, 11), to the 2018 first-in-human study of benign prostatic enlargement (25 participants) (7), the 2022 THERESA liver feasibility study (8 participants) (8), the 2023 U.S. FDA *De Novo* classification of the Edison System (9), and the 2024 #HOPE4LIVER pivotal trial (44 patients and 49 tumors) (10). **(B)** At one year, the #HOPE4LIVER cohort comprised 47 patients and 52 tumors, with local control of 63.4% in the prespecified analysis and 90.0% in a *post hoc* analysis (60). Post-market safety signals are presented with their original denominators: overall complications, 12/230 (5.2%); major complications, 3/230 (1.3%) (61); vascular injury, 4/139 (2.9%) (16); HAKI, 9/146 procedures (6.2%) (64); and PVT, 8/21 (38%) in an enriched high-risk perivascular cohort (17). The three major complications in the 230-patient safety cohort were study-classified deaths attributed to disease progression in patients treated with palliative intent; causality to the histotripsy device or procedure was not established (61). These estimates derive from distinct cohorts and should not be pooled or interpreted as general incidence rates. No randomized head-to-head oncologic trial is currently available (66). FDA, U.S. Food and Drug Administration; HAKI, histotripsy-associated acute kidney injury; PVT, portal vein thrombosis.

### 6.3 Extrahepatic trials

GANNON is a prospective multicenter, single-arm pancreatic feasibility study enrolling up to 50 participants with unresectable locally advanced or oligometastatic pancreatic adenocarcinoma. The record was recruiting and had no results posted at the evidence cutoff (46). CAIN enrolled 20 participants with primary solid renal tumors and is completed, with registry results posted on December 2, 2025 (49). Publicly presented early cohort findings should be described as registry or conference-level evidence until a full peer-reviewed results article becomes available.

### 6.4 Clinical immune endpoints

Clinical immune evidence remains exploratory. A prospective 30-patient liver cohort reported transient cytokine changes and a four-cytokine score associated with one-month response, but the score was derived and tested in the same small cohort and requires independent validation (65). Isolated untreated-lesion responses and circulating biomarker changes are hypothesis-generating. Future studies should prespecify sampling time points, treated tumor fraction, tumor burden, concomitant systemic therapy, and clinical endpoints so that immune correlates can be distinguished from nonspecific inflammation or disease kinetics.

## 7 Discussion: challenges and future directions

### 7.1 Dosimetry and evaluation framework

A useful dosimetry framework links five levels: delivered acoustic input; *in situ* bubble activity; immediate tissue disruption; imaging or acoustic surrogates; and delayed oncologic or immune outcomes. No single surrogate currently validates all levels. Bubble-cloud visualization confirms activity, but not necessarily complete cellular destruction; backscatter, color-Doppler, and acoustic-emission metrics correlate with selected histologic endpoints but remain tissue- and system-dependent (18, 19, 21).

Future studies should report technical success, technique efficacy, local tumor control, retreatment, complications using a stated grading system, procedure duration separated into setup/targeting/sonication, and patient-centered outcomes. For immunology, minimum endpoints should include timing, local versus systemic compartment, assay methods, and linkage to tumor response. Cross-study comparison without these definitions is unreliable.

### 7.2 Patient selection, implementation, and evidence maturity

Patient selection should incorporate acoustic accessibility, target depth, respiratory motion, tumor and intended treatment-zone volume, prior biliary intervention, proximity to large vessels, renal reserve, anticoagulation, and whether complete or partial treatment is intended. Representative treatment times and precision metrics should always be reported with target volume and workflow context; they are not platform-wide constants.

A 2026 meta-analysis of ten studies and 553 patients estimated pooled technical success at 94.1% and major complications at 7.0%, but complication heterogeneity was high and radiologic definitions varied (66). This evidence supports continued prospective development, not definitive comparative effectiveness. Registries, standardized imaging criteria, independent adjudication, and controlled trials are required before histotripsy can be positioned beyond selected liver indications.

## 8 Conclusion

Histotripsy comprises several related but technically distinct focused-ultrasound strategies. Shock-scattering histotripsy, intrinsic threshold histotripsy, and boiling histotripsy should be identified explicitly because waveform, bubble dynamics, dose, monitoring, and biological effects differ. Cavitation threshold-gating helps explain spatial confinement, while tissue mechanics and accumulated exposure shape the final lesion and prevent any assumption of universal vessel or duct preservation.

Preclinical data support DAMP release, immune remodeling, and combination strategies, but immune effects vary by subtype, dose, model, and sampling time and remain unvalidated as a clinical therapeutic mechanism. Early liver studies demonstrate feasibility and promising local control, while post-market evidence has also identified targeting limitations and uncommon but important vascular, thrombotic, and renal complications. Histotripsy should therefore be viewed as an emerging complementary local therapy whose long-term role depends on standardized dosimetry, transparent adverse-event reporting, and prospective comparative evidence.
